# Antiproliferative activity and induction of apoptosis by *Annona muricata* (Annonaceae) extract on human cancer cells

**DOI:** 10.1186/1472-6882-14-516

**Published:** 2014-12-24

**Authors:** Constant Anatole Pieme, Santosh Guru Kumar, Mireille Sylviane Dongmo, Bruno Moukette Moukette, Fabrice Fekam Boyoum, Jeanne Yonkeu Ngogang, Ajit Kumar Saxena

**Affiliations:** Department of Physiological Sciences and Biochemistry, Faculty of Medicine and Biomedical Sciences, University of Yaoundé I, PO Box 1364, Yaoundé, Cameroon; Cancer Pharmacology Division, Indian Institute of Integrative Medicine, 180001, Canal Road, 18001 Jammu, India; Department of Biochemistry and Molecular Biology, University of Buea, Buea, Cameroon; Department of Biochemistry, Faculty of Sciences, University of Yaoundé I, PO Box 812, Yaoundé, Cameroon

**Keywords:** Apoptosis, Membrane mitochondrial potential, Antiproliferative, Cell cycle, A. muricata

## Abstract

**Background:**

*Annona muricata (A. muricata)* is widely distributed in Asia, Africa and South America. Different parts of this plant are used to treat several diseases in Cameroon. The aim of this study is to determine the *in vitro* anti-proliferative effects and apoptotic events of *A. muricata* extracts on HL-60 cells as well as to quantify its phenols content.

**Methods:**

The cell viability was measured by using 3-(4, 5-dimethylthiazol-2-yl)-2, 5-diphenyltetrazolium bromide (MTT) assay while the changes in morphology of HL-60 cells, membrane mitochondrial potential (MMP) and the cell cycle were used for assessment apoptosis induction.

**Results:**

The results show that the concentration of phenols, flavonoids and flavonols in the extracts varied depending on the part of the plant. All the extracts tested inhibited the proliferation of HL-60 cells in a concentration dependent manner with IC_50_ varied from 6–49 μg/mL. The growth inhibition of the cells by extracts was associated with the disruption of MMP, reactive oxygen species (ROS) generation and the G0/G1 cell arrest.

**Conclusion:**

These findings suggest that the extracts from *A. muricata* have strong antiproliferation potential and can induce apoptosis through loss of MMP and G0/G1 phase cell arrest.

## Background

Chemopreventive properties have long been attributed to polyphenolic compounds present in the human diet. The interest on these natural substances is increasing because of their higher potential sources of anticancer compounds. Plants and plant-based medicaments are used as the basis of many modern pharmaceuticals industries today for the treatment of our various ailments [1]. According to world health organization (WHO), more than 80% of the total world’s population depends on the traditional medicines to satisfy their primary health care needs. Several phytochemical molecules from natural products capable of exerting a physiologic action on the human body were studied and characterized. These bioactive compounds such as alkaloids, flavonoids, tannins and phenols were considered to be most important. The phytochemical research that has been done based on the ethno pharmacological information forms the effective approach in the discovery of new anti-infective agents from higher plants [2].

*Annona muricata* L *(A. muricata*) commonly known as graviola, soursop or corossol, belongs to the Annonaceae family. It is a widespread small tree and has its native in Central America [3]. It is a typical tropical tree with heart shaped edible fruits and widely distributed in most of tropical countries. All parts of *A. muricata* tree are used in natural medicine in the tropic including the twigs, leaf, root, fruit and seeds. Generally, the fruit and fruit juice are taken to eliminate worms and parasites, cool fever, increase mother’s milk after child birth, and as an astringent for diarrhea and dysentery [4]. The crushed seeds are used against internal and external parasites, head lice and warms. The twigs, leaf are considered sedative and antispasmodic [4]. A decoction of *A. muricata* leaf is used to kill bed bug and head lice to reduce fever. For the latter it can have the same effect taken orally or added to bathing water [5]. The creamy and delectable flesh of the fruit consist of 80% water, 1% protein, 18% carbohydrates and fair amount of vitamins B, B2 and C, potassium and dietary fiber [6]. The leaf are lanceolate with glossy and dark green in color had been traditionally used to treat headaches, hypertension, cough, asthma and used as antispasmodic, sedative and nervine for heart condition [7, 8]. Previous reports have demonstrated that the leaf, twigs, root, stem, and fruit seed extracts of *A. muricata* have several biological activities such as anti-bacterial [9], antifungal [10] and anti-malarial [11]. Its leaf extract were also found to possess antioxidant [12] and molluscicidal properties [13]. Recently, it has also been reported to exhibit anti-inflammatory and analgesic effects [14], cytotoxicity and apoptosis inducing activities on T47D breast cancer [15], antiviral activity [16] and antidiabetic activity. Phytochemical investigation of the leaf of *A. muricata* showed the presence of alkaloids [17], essential oils [18] and acetogenins [19]. These acetogenins demonstrated to be selectively toxic against various types of the cancerous cells without harming healthy cells [20]. Acetogenin 1 was reported to exhibit cytotoxic activities against the human pancreatic tumor cell line (PACA-2), human prostate adenocarcinoma (PC-3) and human lung carcinoma (A-549), while Acetogenin 2 was reported to exhibit cytotoxicity against human hepatoma carcinoma cell line (Hep G2) [21]. Seven isoquinoline alkaloids including reticuline, coclaurine, coreximine, atherosperminine, stepharine, anomurine and anomuricine have been isolated from the leaves, root and stem barks of *A. muricata* [22]. The essential oil of the fresh fruit pulp of *A. muricata* contains 2-hexenoic acid methyl ester (23.9%), 2-hexenoic acid ethyl ester (8.6%), 2-octenoic acid methyl ester (5.4%), 2-butenoic acid methyl ester (2.4%), β-caryophyllene (12.7%), 1,8-cineole (9.9%), linalool (7.8%), α-terpineol (2.8%), lialyl propionate (2.2%) and calarence (2.2%) [23]. Therefore, we attempted to investigate the growth-inhibitory and apoptotic effects of extracts from leaf, twigs and roots from *A. muricata* against Human promyelocytic leukemia (HL-60 cells).

## Methods

### Preparation of extracts

The plant material including leaf, twigs and roots of were collected in Yaounde, capital city of Cameroon and authentified by Mr NANA, a botanist of the National Herbarium of Cameroon in comparison to the voucher specimens under the reference number of 3289/HNC. They were then shade dried and grounded using a blender. The obtained powder was cold macerated with ethanol 95°C with occasional stirring for 3 days. After 3 days, the suspension was filtered through filtered paper and the filtrate was evaporated to dryness at low temperature, using a rotary evaporator. The procedure was repeated 3 times and until total decoloration of the mixture observed. The obtained extracts were stored and used for further analysis.

### Determination of phenolic composition of the extracts

#### Total phenol determination

The total phenol was determined by the Folin–Ciocalteu method, the reaction mixture contains: 200 μL of diluted spice extract, 800 μL of freshly prepared diluted Folin Ciocalteu reagent and 2 mL of 7.5% sodium carbonate. The final mixture will be diluted to 7 mL with deionized water. Mixture was kept in the dark at ambient conditions for 2 h to complete the reaction. The absorbance at 765 nm will be measured. Garlic acid was used as standard and the results were expressed as mg garlic acid (GAE)/g of dried material.

#### Determination of total flavonoid content

Total flavonoid content was determined using aluminium chloride (AlCl_3_) according to a known method using quercetin as a standard. The spice extract (0.1 mL) was added to 0.3 mL distilled water followed by 5% NaNO_2_ (0.03 mL). After 5 min at 25°C, AlCl_3_ (0.03 mL, 10%) was added. After further 5 min, the reaction mixture was treated with 0.2 mL of 1 mM NaOH. Finally, the reaction mixture was diluted to 1 mL with water and the absorbance was measured at 510 nm. The results will be expressed as mg of quercetin (QE)/g of dried material

#### Determination of total flavonols

Total flavonols in the plant extracts were estimated using the method of Kumaran and Karunakaran [24]. To 2.0 mL of sample (standard), 2.0 mL of 2% AlCl_3_ ethanol and 3.0 mL (50 g/L) sodium acetate solutions were added. The absorption at 440 nm was read after 2.5 h at 20°C. Extract samples were evaluated at a final concentration of 0.1 mg/mL. Total flavonoid content was calculated as mg of quercetin (mg/g) using the following equation y = 5.3911x + 0.0313, R^2^ = 0.9967 based on the calibration curve, where x was the absorbance and y was the concentration of quercetin (mg/g).

### Antitumor activity of the extracts

#### Cell culture

The Human promyelocytic leukemia (HL-60 cells) was obtained from European Collection of Cells Culture (ECCC), Sigma–Aldrich, India. They were grown in RPMI-1640 medium containing 10% Fetal bovine serum (FBS), penicillin (100 IU/mL) and streptomycin (100 μg/mL medium). The cells suspension was kept in the incubator (Thermo Electron Corporation, USA) at 37°C, 5% CO_2_; 95% humidity. Cells were used for different assays during logarithmic growth phase while the untreated control cultures received only the vehicle (dimethyl sulfoxide [DMSO] < 0.1%).

#### Cells viability and treatments

HL-60 cells were seeded in 96 different well plates containing 15 × 10^3^ μL/well, respectively. The cultured cells were then treated (triplicate wells per condition) by adding of 100 μL of serial dilutions of the three extracts in DMSO to give a final concentration of 100, 30, 10 and 1 μg/mL. In addition, the DMSO alone was added to another set of cells as the solvent control (DMSO < 0.1%). The cells were then incubated for another 48 h prior to the addition of 20 μL of 2.5 mg/mL solution of 3-(4, 5-dimethylthiazol-2-yl)-2, 5-diphenyltetrazolium bromide (MTT) into each well. The incubation was continued for 3 h before the media was removed. A mixture of DMSO (150 μL) was added to each well and mixed to ensure dissolving of the crystal formazan before the absorbance at 570 nm was measured. Three replications of each experiment were performed and fifty percent of inhibitory concentration (IC_50_) of each extract was calculated.

#### Hoechst 33258 staining of cells for nuclear morphology

HL-60 cells (2×10^6^ cells/3 mL/well) were treated with different extracts at different concentrations of extract for 24 h. They were collected, centrifuged at 400 g and washed once with PBS. A solution of Hoechst (Hoechst, 10 μg/mL; citric 10 mM; Na_2_HPO_4_ 0.45 M; Tween-20 0.05%) was added in each tube and kept in the dark at room temperature for 30 min. The mixture was washed with PBS and the pellet suspended in 100 μL of PBS/glycerol (1:1). The solution (10 μL) was poured into the slide and nuclear morphology alterations observed under fluorescence microscope (Olympus X 70, magnification 20 X) [25].

#### Reactive oxygen species (ROS) assay

ROS production was monitored by flow cytometry using 2’ , 7’- dichlorodihydrofluorescin diacetate (DCFH_2_-DA). This dye is a stable non polar compound that readily diffuses into cells and is hydrolyzed by intracellular esterase to yield 2’ ,7’ dichlorodihydrofluorescin (DCFH_2_), which is trapped within the cells. Hydrogen peroxide or low molecular weight peroxides produced by the cells oxidizes DCFH_2_ to a highly fluorescent compound 2’ ,7’-dichlorofluorescein (DCF). Thus, the fluorescence intensity was proportional to the amount of hydrogen peroxide produced by the cells. Briefly, HL-60 cells (1 × 10^6^ cells/2 mL/well) were treated with extracts of leaf, roots and twigs of *A. muricata* at different concentrations for 24 h. Thirty minutes before the end of the experiment, the cell culture was treated with DCFH_2_-DA (50 μM) and kept in the dark. Cells were then collected, centrifuged (200 g; 4°C; 5 min) and the pellet was washed with 1 mL of PBS and centrifuged as mentioned earlier. The pellet was suspended in 500 μL of PBS and the fluorescence was assessed by comparing two fluorescence emission 480 nm/530 nm using a flow-cytometer (BD-LSR).

#### Mitochondrial membrane potential (MMP) assay

HL-60 cells (1×10^6^ cells/2 mL/well) were treated with the three extracts at different concentrations for 24 h. Thirty minutes before the end of the experiment, the cell culture was treated with Rhodamine-123 (200nM) and kept in the dark for 30 mn. Cells were collected, centrifuged (400 g; 4°C; 5 min), the pellet was washed with 1 mL of PBS and centrifuged as mentioned earlier. The depolarization of mitochondrial membrane was examined by measuring the fluorescence emission shift (red to green) of the Δψm sensitive cationic Rh-123 dye. The fluorescence intensity of 10,000 events was analyzed in FL-1 channel on BD FACS Calibur (Becton Dickinson, USA) flow cytometer. The decrease in fluorescence intensity because of MMP loss was analyzed in FL-1 channel and the change of potential membrane (Δψm) was assessed by comparing fluorescence.

#### DNA content and cell cycle phase distribution

HL-60 cells (1×10^6^ cells/2 mL/well) were treated with extracts at 20, 50, 100 μg/mL for 24 h. They were harvested and washed with 1 mL of PBS, then centrifuged at 400 g for 5 min at 4°C. The pellet was suspended in 100 μL of PBS and 900 μL of hypertonic buffer (PI-25 μg/mL, RNAase- 40 μg/mL, sodium citrate-0.1% and Triton-100X-0.03%) and incubated at 37°C in dark for 20 min. Finally, cells were analyzed immediately on flow cytometer FACS Calibur (Becton Dickinson, USA). The data were collected in list mode on 10,000 events and illustrated in a histogram, where the number of cells (counts) is plotted against the relative fluorescence intensity of PI (FL-2; λem: 585 nm; red fluorescence). The resulting DNA distributions were analyzed by Modfit (Verity Software House Inc., Topsham, ME) for the proportions of cells in G0/G1, S phase, and G2/M phases of the cell cycle [26].

### Statistical analysis

The viability experiments were done in triplicates and each data point represents the average of at least 3 independent experiments. The distributions of the data are abnormal. The data was expressed as mean ± SD. In order to carry out statistical analysis, the data was analyzed using SPSS (Version 11.5; SPSS Inc.,) and M.S. Office, Excel software. One way analysis of variance technique was applied to observe the significance between the groups. The post hoc test Duncan’s multiple range test was performed to know the significant difference among the groups. Entire statistical analysis was carried out at p < 0.05.

## Results

### Phenolic contents of *A. muricata*

The results of this study showed that the level of polyphenols, flavonoids and flavonols varied depending on the part of the plant (Table 1). The concentration of these three groups of molecules is higher in the leaves compare other parts of plant extract. The lowest concentration of flavonoids and flavonols was found on the stem barks of *A. muricata* while its roots show the lower phenols content.

**Table 1:** **Phenolic composition and fifty percent inhibition of extracts of** ***A. murica***

Parts of plant used	HL-60 cells IC50 (μg/mL)	Total polyphenol (mg/g dried material)	Flavonoids (mg/g dried material)	Flavonols (mg/g dried material)
T	49 ± 3.2^b^	19.88 ± 1.52^b^	6.03 ± 0.92^b^	2.40 ± 0.90^b^
R	9 ± 0.8^a^	13.01 ± 0.58^c^	6.82 ± 1.35^b^	3.63 ± 2.81^b^
L	14 ± 2.4^b^	23.07 ± 1.56^a^	9.96 ± 1.53^a^	6.57 ± 1.42^a^

Values are represented as mean ± SD, n = 3; L: Leaf; R: Roots; T: twigs, Values affected with different letters are significantly different (p < 0.05).

### *In vitro*anticancer activity

#### Effects of A. muricata extracts on the proliferation of HL-60

**Figure 1:**
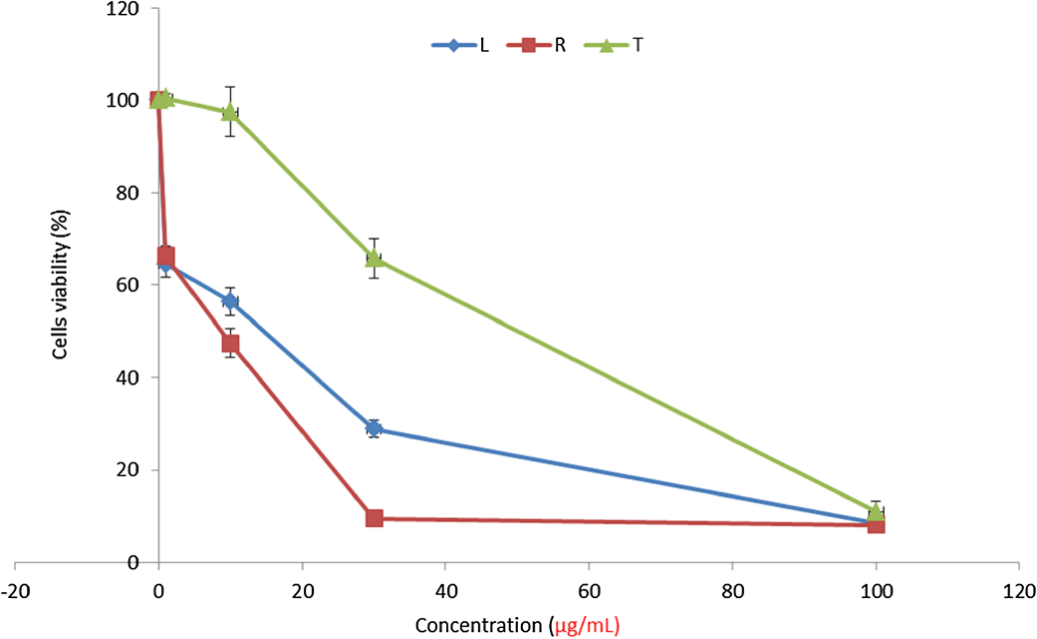
**Viability of HL-60 cells after 48 h treatment with extracts of** ***A. muricata*** **; (n = 3); (A) HL-60 cells; L (Leaf); R (Roots); T (twigs).**

In this study we used a microculture assay based on metabolic reduction of MTT to evaluate the cytotoxic effect extracts of *A. muricata* on HL-60 cells. This technique permitted us to evaluate dose-dependent effect, by linear regression analysis showing acceptable R^2^ values and correlation coefficients. As shown in the Figure 1, the addition of extracts at different concentration to the cultured cells inhibited dramatically and significantly the proliferation of the cells in a dose-dependent manner. The values of IC_50_ of the extract after 48 h was between 6–12 μg/mL which is lower than 20 μg/mL (Table 1).

#### Morphological changes of apoptotic treated HL-60 cells with A. muricata extracts

**Figure 2:**
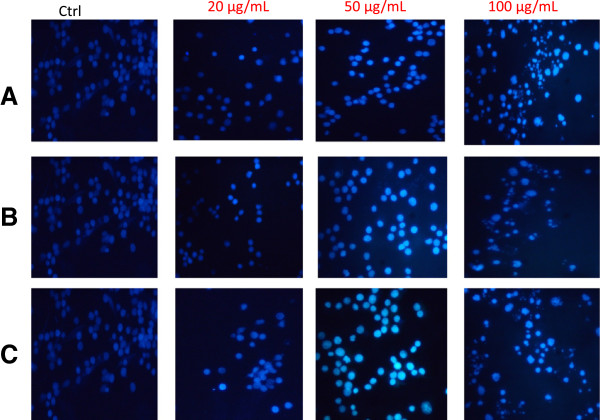
**Effect of extracts** ***A. muricata*** **on nuclear morphological changes of HL-60 cells.** After 24 h of treatment cells staining with Hoechst 33258 incubated for 30 min, and observed under fluorescence microscope. Olympus,Tokyo, Japan; magnification 200×. Marked morphological changes of cell apoptosis such as condensation of chromatin and nuclear fragmentations were found clearly. Apoptotic cells gradually increased in a dose-dependent manner; **(A)**: Leaf; **(B)**: Roots; **(C)**: twigs.

To investigate of *A. muricata* extracts on the nuclear modification on HL-60 cells, the Hoechst 33258 staining test was performed at different concentrations (20, 50 and 100 μg/mL) after 24 h of treatment. Hoechst 33258 reagent is a membrane-permeable blue fluorescent dye which stained cell nucleus. As observed in Figure 2, the control or untreated cells present the characteristics of healthy cells. They appeared to be intact oval shape and the uniform nuclei were stained with a less bright blue fluorescence. Cells treated with tested extracts exhibited a bright blue color when the concentration of extract increases. These results demonstrated typical features of apoptosis such as cell shrinkage, chromatin condensation, and fragmentation to multiple aggregate of apoptotic bodies and cell decrement (Figure 2). The apoptotic nuclei clearly showed highly condensed or fragmented chromatin. At 100 μg/mL, most of the cells undergo apoptosis and the number of apoptotic bodies increases.

#### A. muricata extracts induce apoptosis by generation of ROS

**Figure 3:**
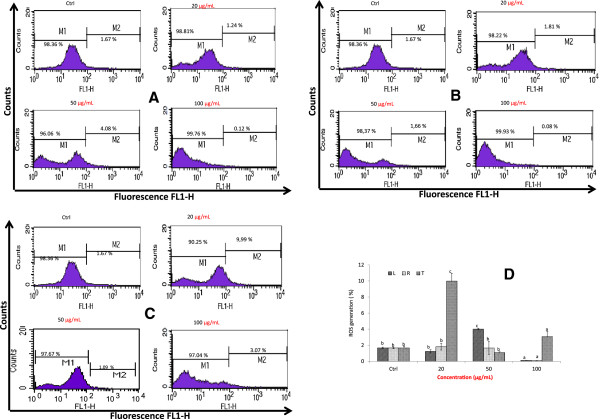
**Effects of extracts** ***A. muricata*** **on ROS production on HL-60 cells; cells were treated with extract for 24 h followed by staining with DCHFH**_**2**_ **-DA (50 μM), incubated for 30 min and the fluorescence in the cells was immediately analyzed using flow cytometry.** Data are presented the fluorescence intensity; **(A)**: Leaf; **(B)**: Roots; **(C)**: twigs: **(D)**; Variation of ROS production of extracts of *A. muricata*; values are expressed as means ± standard error (n = 3). Values affected with different letters are significantly different (p < 0.05) from the control. L: Leaf; R: Roots; T: twigs, Ctl: control; app: apoptic phase.

To investigate whether extracts of *A. muricata* inhibit the HL-60 cells by through the generation of ROS, we monitored the redox status of the HL-60 treated cells using the oxidation sensitive fluorescent dye DCFDA. As shown in Figure 3A, B and C, the ROS levels generated after 24 h treatment of HL-60 cells varied with the concentration of extract and the part of plant used in the study. However, the higher ROS levels were found at the concentration of 20 μg/mL (9.99% for twigs), 50 μg/mL (4.08% for Leaf) and 100 μg/mL (3.07% for the twigs) (Figure 3D). At the higher concentration (100 μg/mL), the ROS produced by cells with the extract of the roots and leaves were lower than that of untreated control cells (Figure 3D).

#### A. muricata extracts disrupt mitochondrial membrane potential in HL-60 cells

**Figure 4:**
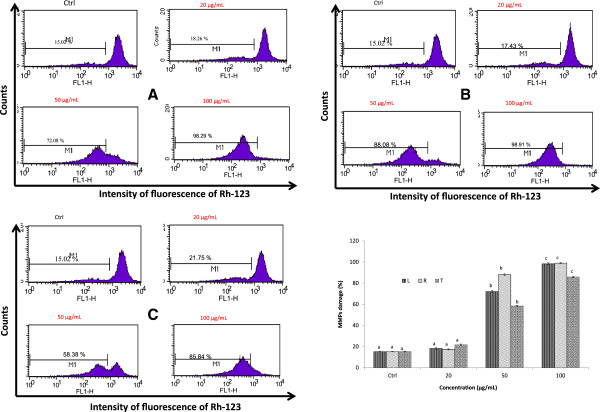
**Effects of extracts of** ***A. muricata*** **on the integrity of mitochondrial membrane, HL-60 cells were treated different concentrations of extracts, incubated 1 h with 200 nM of Rh-123 and then analyzed by flow cytometry.** Data are presented the fluorescence intensity: **(A)**: Leaf; **(B)**: Roots; **(C)**: twigs: **(D)**; Variation of the integrity of mitochondrial membrane by extracts of *A. muricata*; values are expressed as means ± standard error (n = 3). Values affected with different letters are significantly different (p < 0.05) from the control. L: Leaf; R: Roots; T: twigs; Ctl: control.

After treatment of cells with extracts at different concentration, we observed an increase of fluorescence intensity indicating the mitochondrial membrane depolarization as Figure 4A, B & C. The depolarization of mitochondrial membrane varied with concentration and the extracts. A dose-dependent increase of fluorescence observed is ranging from 17.34% to 98.91% (Figure 4B). Among the extract tested, the twigs extract demonstrated a higher depolarization of mitochondrial membrane (21.75%) at 20 μg/mL while those of leaf and roots showed a maximum depolarization at 100 μg/mL (98.29 and 98.91% respectively). All the extracts at 50 μg/mL exhibited more than 50% of depolarization of mitochondrial membrane (Figure 4D). These results show that extracts induced apoptosis on HL-60 cells after 24 h through the disruption of mitochondrial membrane.

#### A. muricata extracts induce a G0/G1 cell cycle arrest in HL-60 cells

**Figure 5:**
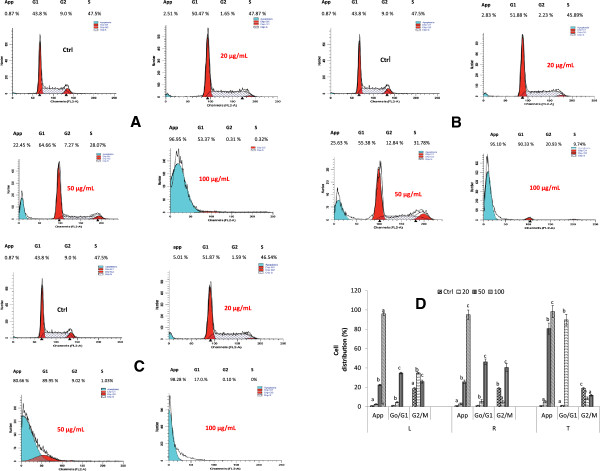
**Cell cycle analysis of extracts of** ***A. muricata*** **on HL-60 cells.** After 24 h, treated cells were incubated with RNAse (40 μg/mL), stained with propidium iodide (25 μg/mL), and analyzed by BD-FACS Caliber flow-cytometer; **(A)**: Leaf; **(B)**: Roots; **(C)**: twigs; Cells distribution after treatment with extracts of *A. muricata*; values are expressed as means ± standard error (n = 3). Values affected with different letters are significantly different (p < 0.05) from the control. L: Leaf; R: Roots; T: twigs; Ctl: control; app: apoptic phase.

The changes in the cell cycle distribution were shown in Figure 5A and the apoptotic cells were counted based on G0 DNA contents. The results show no changes in the cell cycle distribution of the control group, however, the accumulation of cells was found in apoptotic (G0) with significant modification of G2/M and S phases when the concentration of all the tested extracts increase (Figure 5A, B & C). The cell population of G0 phase significantly increased from 2.51 – 96% (leaf), 2.83 – 95% (roots) and 5.01 – 98% (twigs) and in the meantime the proportion G0/G1drastically increased (Figure 5D). The results demonstrated that all the tested extracts induced apoptosis on HL-60 cells through G0/G1 phase cell cycle arrest.

## Discussion

Natural polyphenols are secondary metabolites produced by plants for their defense against different types of stress, e.g. ultraviolet radiation, aggression of pathogens, low soil fertility, changes of environmental temperature, severe drought, and grazing pressure [27]. The interest on plant phenols is increasing in the recent decade because of their health promoting potential. It is widely known that diets containing an abundance of phenols have protective effects against a variety of diseases, particularly cardiovascular disease and cancer. Phenols from herbal extract are raising great interest as powerful and safe anticancer strategy for their broad range targeting capability and low side effects. Depending on the chemical structure, several beneficial effects of polyphenols and their implications in the human health have been identified including in cancer [28], neuroprotection [6, 29], cardiovascular system dysfunction and damage, the metabolic syndrome, diabetes, aging, and different inflammation-related pathologies [27, 30, 31]. Chemotherapy drugs from polyphenols could improve the survival of cancer patients, with low side effects. Thus, it is urgent to develop novel drugs which are more effective [32]. Novel strategies for determination of natural products with biological activity require the implementation of large-scale screening programs.

The antiproliferative activities of *A. muricata* have been carried out on several cancer cells with significant positive results [15, 21]. Plant extracts with IC_50_ values ≤ 30 μg/mL are considered pharmaceutically active [33]. Our MTT results (Table 1) indicated that extracts of *A. muricata* inhibited significantly the HL-60 cells *in vitro* and can be considered as active according to the suggestions from the National Cancer Institute (NCI) which stated that the IC_50_ lower or equal to 20 μg/mL can be used as a benchmark for suitable screening cancer drugs from plants and herbs [34]. A dose- dependent inhibitory effect of extracts was also observed in HL-60 treated cells (Figure 1A). Among these extracts the roots exhibited the higher cytotoxic effects than other extracts (Table 1). Our results demonstrated that *A. muricata* extracts have significant cytotoxic potential on HL-60 cells.

Apoptosis is a crucial mode of programmed cell death, which is an active physiological process to eliminate selectively unnecessary cells [35]. Induction of cell apoptosis in tumor tissue is the best stage for cancer therapy [35]. Apoptosis is a common mode of action of chemotherapeutic agents, including the natural product-derived drugs. Furthermore, induction of apoptosis is recognized as an efficient strategy for cancer chemotherapy and a useful indicator for cancer treatment and prevention. Hence several researchers nowadays have performed apoptotic screening of natural products from herbal extracts in Cameroon [36, 37]. Apoptosis involves specific morphological and biochemical changes such as chromatin condensation, membrane blebbing, cell shrinkage, DNA fragmentation, etc. Induction of apoptosis is the key to success of plant products as anticancer agents [38, 39]. After 24 h of treatment with *A. muricata* extracts, the characteristics of apoptotic cells, including the increase of bright blue color of Hoechst 33258 staining was observed (Figure 2A, B & C) as well as evident DNA fragmentations at 100 μg/mL, which are the important hallmarks of apoptosis [40]. The results indicated that all the extracts from *A. muricata* induce apoptosis of HL-60 cells.

Mitochondrion is an integral part of apoptotic machinery and events such as loss of mitochondrial membrane potential is classical evidence for apoptosis [35]. Usually, the decrease in MMP occurs during the early stage of apoptosis before the cell morphology changes. The sharp decrease in the membrane potential indicates the irreversible occurrence of early apoptosis due to an increase in the permeability of the mitochondrial membrane follow by the release of apoptotic factors, including cytochrome c [41]. Based on the current research, we propose that the following mechanism is responsible for extracts-induced HL-60 cell apoptosis. Briefly, extracts of *A. muricata* acts through the disruption of membrane mitochondrial to arrest cells in the G0/G1 phase and inhibit cell proliferation. Cell cycle checkpoints are control mechanisms that ensure the proper progression of cell cycle events [42]. *In vitro* apoptosis assay study presented here (Figure 5A, B, C), showed that extracts from *A. muricata* induced apoptosis in HL-60 tumor cells in a dose-dependent manner, compared to untreated cells. In this experiment, the extracts of *A. muricata* induced G0/G1 cell cycle arrest in HL-60 cells at different concentration after 24 h of treatment (Figure 5A, B, C). Therefore, we can suggest that the anticancer effects of *A. muricata* extract is associated with G0/G1 cell cycle arrest and cell differentiation. The cell cycle analysis revealed that all the extracts of *A. muricata* can markedly induce a G0/G1 phase arrest in HL-60 cells, but they have low effects on the G2/M phase. Although the induction mechanism of cell differentiation by *A. muricata* extract is not clear, a block of cell cycle progression at the G0/G1 phase may be closely related to the differentiation.

Most chemopreventive agents known today from plant extracts are subdivided into two categories: (i) blocking agents which inhibit the initiation step by preventing carcinogen activation and (ii) suppressing agents, which inhibit malignant cell proliferation during promotion and progression steps of carcinogenesis [43]. Natural products, including medicinal plants, herbs and spices provide rich resources for anticancer drug discovery [44]. Phytochemical analysis of the extracts of *A. muricata* was performed in order to identify the chemical nature of the active principles. Quantitative analysis of the extracts revealed the presence of phenolics, flavonoids and trace amounts of flavonols (Table 1). Previous studies, demonstrated the presence of a number of phytochemicals, including phenols as flavonoids, tannins, anthraquinones and steroids in the leaf extracts of *A. muricata* [45]. Cinnamic acid derivative, Coumaric acid hexose, 5-Caffeoylquinic acid, Dihydrokaempferol-hexoside, p-Coumaric acid, Caffeic acid derivative and Dicaffeoylquinic acid represented phenolics acids group were isolated in its fruits [46]. Intensive chemical investigations of the leaves and seeds of *A. muricata* have resulted in the isolation and identification of a great number of acetogenins with interesting biological or pharmacological activities, such as antitumoral, cytotoxicity and apoptosis on HCT-116 and HT-29 cancer cells, [16, 47]. Hence, previous studies revealed that several acetogenins act as a DNA topoisomerase I poison, arrested cancer cells at the G1 phase and induced apoptotic cell death in a Bax-and caspase-3- related pathways, and inhibited NADH-ubiquinone oxidoreductase (complex I) in mitochondria [21]. It can be suggested that the synergistic effects of phytochemicals present in this plant extracts including acetogenins may be the underlying principle behind the chemotherapy potential agents of *A. muricata*. These groups of molecules found in a large quantity in *A. muricata* extracts may act in synergy and might be responsible for their antiproliferation activity through the significant decrease in the mitochondrial membrane potential induction of apoptosis.

## Conclusions

In conclusion, *A. muricata* exhibit antiproliferative effects of HL-60 cells by inducing loss of cell viability, morphology changes, loss of membrane mitochondrial potential and G0/G1 phase cell arrest. Our data confirm the potential of *A. muricata* as an agent of chemotherapeutic and cytostatic activity in HL-60 cells. These data suggest that these extracts have potential for cancer chemotherapy. However, the complete mechanisms underlying the therapeutic effects of the extracts need to be investigated as well as the identification of the active molecules present in the different parts of the plant.
